# Evaluation of Mixing Temperature in the Preparation of Plant-Based Bigels

**DOI:** 10.3390/gels10110725

**Published:** 2024-11-09

**Authors:** Marcela Quilaqueo, Sonia Millao, Eduardo Morales, Mónica Rubilar, Ingrid Contardo

**Affiliations:** 1 Department of Chemical Engineering, Faculty of Engineering and Science, Universidad de La Frontera, Temuco 4811230, Chile; sonia.millao@gmail.com (S.M.); eduardo.morales@ufrontera.cl (E.M.); monica.rubilar@ufrontera.cl (M.R.); 2 Scientific and Technological Bioresource Nucleus BIOREN, Universidad de La Frontera, Avenida Francisco Salazar 01145, Temuco 4811230, Chile; 3 Biopolymer Research & Engineering Laboratory (BiopREL), School of Nutrition and Dietetics, Faculty of Medicine, Universidad de los Andes, Chile, Monseñor Álvaro del Portillo 12.455, Las Condes 7620086, Chile; 4 Centro de Investigación e Innovación Biomédica (CIIB), Universidad de los Andes, Monseñor Álvaro del Portillo 12.455, Las Condes 7620086, Chile

**Keywords:** canola oil, carnauba wax, Arabic gum, oleogel, hydrogel, droplet size, hybrid gel

## Abstract

Understanding gel structures and behavior is a prerequisite for attaining the desired food application characteristics. The mixing temperature is crucial when incorporating thermolabile active compounds into gels. This study evaluated the effect of mixing temperature on the physical and chemical properties of a bigel system prepared using a carnauba wax/canola oil oleogel and Arabic gum hydrogels. The results showed that bigels prepared at lower temperatures (30 and 40 °C) resulted in a solid-like state under crystallization temperature, resulting in matrices with larger hydrogel droplets, softer texture, and lower adhesiveness, spreadability, and solvent binding capacity. In contrast, bigels prepared at higher temperatures (50 and 60 °C), around crystallization temperature but with no solid state, resulted in matrices with smaller hydrogel droplets and higher firmness, adhesiveness, and spreadability. These bigels had a higher apparent viscosity, especially at lower shear rates, and solid-like behavior in the linear viscosity range. During the bigel preparation process, adjusting the mixture temperature had no effect on the samples’ oxidative stability, FTIR spectra, or thermal properties. The results highlighted the importance of hydrogel droplet size on the microstructure of the formed bigels, and smaller droplets could act as effective fillers to reinforce the matrix without making chemical changes.

## 1. Introduction

A gel is a substance exhibiting properties that are transitional between a solid and a liquid, demonstrating a blend of elastic (solid) and fluid (liquid) characteristics. Gels include hydrogels (water-based) and oleogels (oils), which have a wide range of applications due to their properties. Food gels are used worldwide to produce various products with desirable attributes, like jams, jellies, confectionery, desserts, yogurt, etc., notably influencing the textural properties [1,2].

Hydrogels are a series of soft and wet materials with low volume-fraction, three-dimensional porous networks of polymer molecules, fibers, or particles in which the water or aqueous phase acts as the dispersion medium. Hydrogels play a critical role in food science, such as in structuring foods with desired sensorial textures, preserving metastable food structures, increasing shelf-life, designing food packaging materials, nutraceutical delivery and bioavailability, calorie control, and risk monitoring for food safety, among others [3]. Hydrogels have notable features, including superior spreadability and miscibility, along with compatibility with various excipients (i.e., solvents), facilitating their application across a wide range of applications [4].

Oleogels are oil-based materials in which edible oil is structured using an oil-structuring agent, usually a lipid-based material. The oleogel structure can be formed using direct or indirect approaches, either by self-assembling low molecular weight molecules in several arrangements or by high molecular weight polymer networks [5]. Different edible oleogels are formulated using various techniques and materials, and they have been used as saturated and trans-fat replacers in spreads, baked goods, confectioneries, and in dairy and meat products [6].

A novel class of material that has attracted attention in food science in recent years is bigels. Bigels are biphasic structures distinguished by their two gelled phases, typically, the oil phase (oleogel) and the aqueous phase (hydrogel). Similar to emulsions, bigels exist as oleogel-in-hydrogel or hydrogel-in-oleogel systems, where the gelled dispersed phases fragment into small droplets, or as bi-continuous bigels that lack a distinct dispersed phase [7]. Bigels have received extensive attention as carriers for drug delivery, especially in transdermal uses. Given the distinctive characteristics and potential of bigels, further research is crucial to broaden their applications in the pharmaceutical and food industries [8]. Properly understanding gel structures and behavior is a prerequisite to attaining the desired application characteristics. The physicochemical characteristics of gelling agents (such as molecular dimension, surface activity, polarity, and thermal stability) and the processing or environmental conditions (such as temperature, pressure/shear, ionic strength, etc.) can result in different types of structures and, consequently, materials with other characteristics. For example, the preparation (or mixing) temperature is very important when incorporating thermolabile active compounds into gels.

Several materials have been studied to prepare food-grade oleogels and hydrogels. Carnauba wax (CW) is a natural wax derived from the leaves of the Brazilian palm Copernicia. It has a high melting point (about 85 °C), low solubility, and appears as a white to grayish-brown powder. It is non-toxic with no chronic effects and demonstrates antifungal and antioxidant qualities. The capacity of carnauba wax to act as a gelling agent in oils to form oleogels as fat replacers is well documented, with good results in different foods [9,10]. On the other hand, canola oil (CO), from a variety of rapeseed, has a minimum percentage of erucic acid and glucosinolates, and is a valuable provider of oleic acid (50–66%), α-linolenic acid (6–14%), and unsaturated fatty acids. Additionally, it contains significant amounts of phytosterols, tocopherols, and polyphenols, which contribute to serum LDL level reduction. Therefore, canola oil has beneficial nutritional characteristics that promote health [11,12]. Arabic gum (AG), an amphiphilic polysaccharide obtained from Acacia Senegal trees, has been extensively studied for the preparation of hydrogels Among all gum exudates, Arabic gum has the highest commercial value due to its widespread application in the food, pharmaceutical, and cosmetic industries [13]. However, the combination of a CW/canola oil oleogel and an AG hydrogel in a bigel system has not been studied.

This research evaluated the effect of mixing temperatures (30, 40, 50, and 60 °C) on the physical and chemical properties of a bigel system prepared using food-grade plant-based materials, CW/CO oleogel, and AG. It is hypothesized that by changing the mixing temperature in the preparation of bigels, the physical properties of a bigel can be modulated.

## 2. Results and Discussion

### 2.1. X-Ray Diffraction

**Figure 1:**
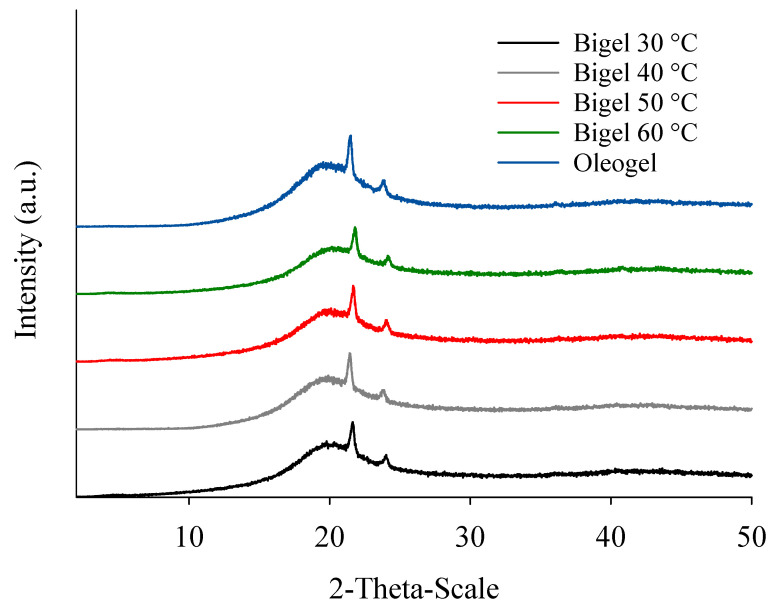
X-ray diffraction patterns of bigels and the oleogel.

The CW/CO oleogel is a semicrystalline solid with a crystalline component of a medium degree and an amorphous component that contributes greatly to the background diffraction pattern. The crystal structure is attributed to the CW, with two separate, intensive, narrow peaks at 4.1 and 3.7 Å, representing B’ and B polymorphs, respectively (Figure 1). On the other hand, the amorphous component, shown by a broad diffraction peak, is attributed to the liquid oil [14]. In the bigel samples, which are semi-crystalline solids, peaks at 4.1 and 3.7 Å can be found but are less intense than in the oleogel due to the dilution effect provoked by the hydrogel. When bigels with the same composition are manufactured at various mixing temperatures, the peaks’ intensities are similar by comparison.

### 2.2. Infrared Spectroscopy

**Figure 2:**
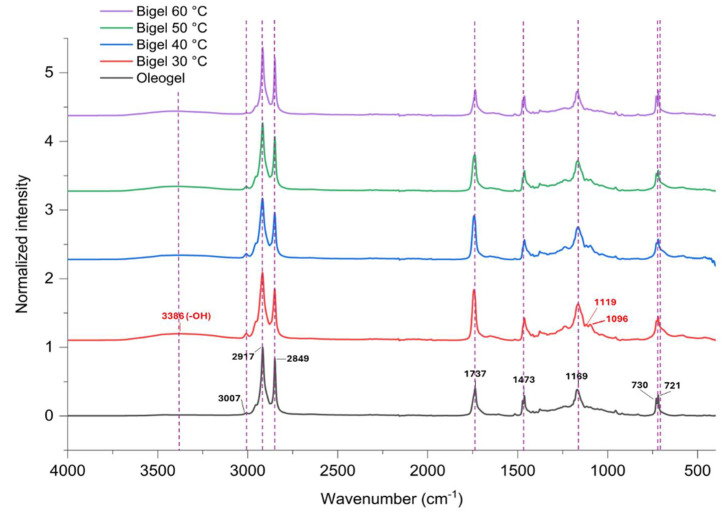
FTIR spectra of oleogel and bigels prepared at different mixing temperatures.

The FTIR spectra of oleogels and bigels are shown in Figure 2. Most of the spectra were observed in two regions (3100–2800 and 1800–1000 cm^−1^), which confirm the presence or absence of specific functional groups. The oleogel spectrum exhibited specific peaks at approximately 2917, 2849, 1737, 1473,1463, 1186, 1169, 730, and 720 cm^−1^. The peaks at 2917 and 1737 cm^−1^ confirmed the presence of C–H stretching (CH2 group) and C=O stretching (ester carbonyl functional groups of the triglycerides), respectively, which are the characteristic peaks of CO and CW [15,16]. The small band at 3007 cm^−1^ was assigned to C–H stretching (symmetric vibration of the cis double bonds, =CH) and could indicate the presence of unsaturated fatty acids in vegetable oils. The IR spectrum of the bigels was more like that of the oleogel, because there is more canola oil than AG in the bigels. However, some differences in the fingerprint region were associated with hydrogel incorporation. The appearance of peaks in the 1095 to 1120 cm^−1^ range for all bigels confirmed the presence of glycosidic linkages in the AG. Likewise, the small broad peaks at approximately 3386 cm^−1^ may indicate the contribution of –OH stretching vibrations of the AG [17]. No major shifts or new peaks from interactions between the components of both phases were identified when the homogenization temperature was increased from 30 to 60 °C, indicating the physical arrangement of the bigels [18].

### 2.3. Differential Scanning Calorimeter (DSC)

**Figure 3:**
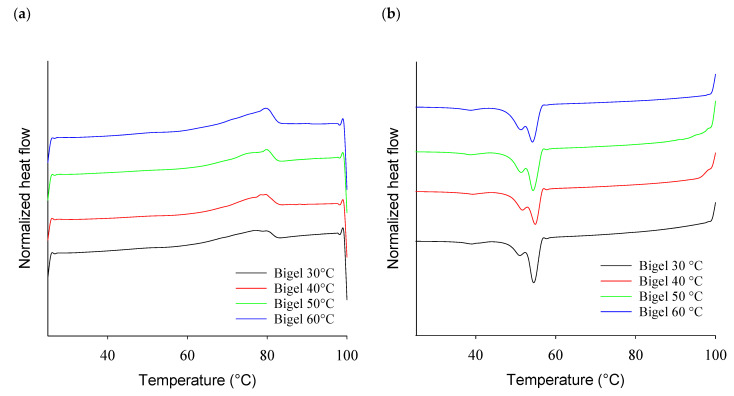
Normalized thermograms for DCS of bigels prepared at different mixing temperatures for heating (**a**) and cooling (**b**).

The thermal properties, expressed as peak melting and crystallization temperatures, showed that the mixing temperature did not significantly affect the bigels (Table 1, Figure 3). In the melting behavior, two peaks were observed in the bigels and the oleogel thermograms (at 49–50 °C and 76–80 °C), and all bigels showed peaks at temperatures similar to the oleogel. In the crystallization curves, three peaks were found (54, 50–53, and 54 °C), and the second peak of all the bigels was (statistically) significantly lower than in the oleogel, but this difference was just about 2 °C.

### 2.4. Optical and Confocal Laser Scanning Microscopy

**Figure 4:**
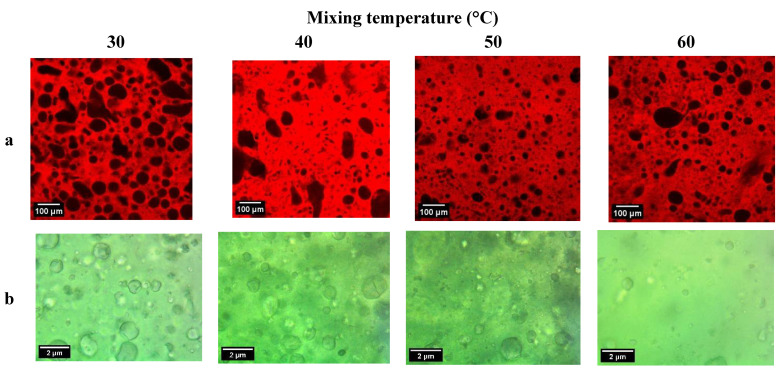
Confocal (**a**) and optical (**b**) microscopy images of bigels prepared with carnauba wax/canola oil oleogel and Arabic gum hydrogel at different mixing temperatures.

Confocal microscopy showed that all bigels had a water-in-oil (hydrogel-in-oleogel) phase distribution (Figure 4). From qualitative analyses, samples prepared at 30 °C and 40 °C showed evidence of larger droplets in the aqueous phase; in bigels prepared at 30 °C, there was a homogeneous droplet size distribution in the matrix, while in bigels prepared at 40 °C, droplets of different sizes were observed. In bigels prepared at 50 and 60 °C, the observed dispersed droplets were smaller. These results coincide with what was found using an optical microscope; as the mixing temperature increased, the size of the droplets observed decreased.

### 2.5. Solvent Binding Capacity (SBC)

**Figure 5:**
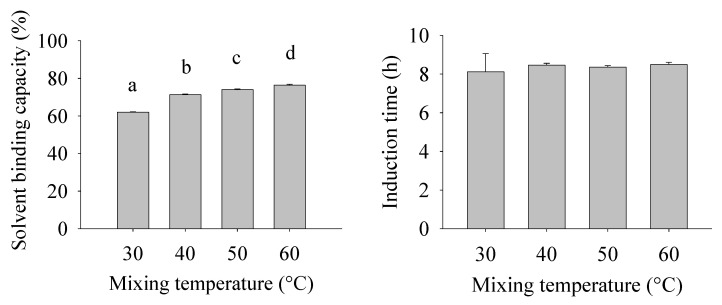
Solvent binding capacity and oxidative stability of bigels prepared at different mixing temperatures. The lower-case letter a-d indicate significant differences according to ANOVA and Tukey’s test (*p* < 0.05).

The SBC values of bigels range between 62.6% and 76.3%, and significant differences were found among all samples (Figure 5). The mixing temperature showed a positive correlation with the SBC (r = 0.9414) of bigels; as the mixing temperature increases, the SBC increases. A possible explanation is that higher mixing temperatures allow the formation of smaller hydrogel droplets, dispersed in the oleogel matrix, (as is observed in Figure 3) that can act as an effective filler, giving better stability in the matrix. At higher mixing temperatures, the oleogel is still like a semi-liquid mixture of wax and oil because it is over the first melting peak of the bigels, as is shown in the DSC analyses (Table 1), favoring the formation of smaller hydrogel droplets. The SBC should be high enough to ensure no oil or water separation in applications [19]; thus, a higher mixing temperature favors the formation of a structure that is able to improve the liquid entrapment.

### 2.6. Texture Determination

**Figure 6:**
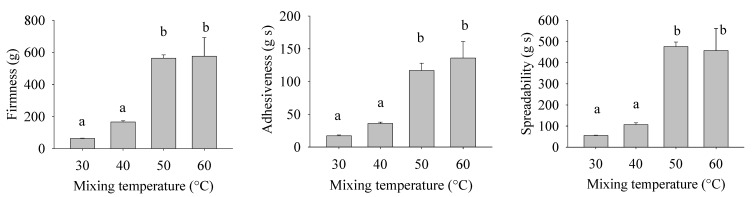
Textural parameters of bigels prepared at different mixing temperatures. The lower-case letter a-b indicate significant differences according to ANOVA and Tukey’s test (*p* < 0.05).

A texture analysis was conducted to ascertain the influence of mixing temperature on the mechanical properties of the bigel (Figure 6). First, the firmness increases significantly (*p* < 0.05) with the increase in mixing temperature, especially from 40 to 50 °C; at 50 °C, the firmness was three times the firmness at 40 °C. However, no significant differences (*p* > 0.05) were found in samples at mixing temperatures between 30 and 40 °C and between 50 and 60 °C. A significant positive correlation (r = 0.9398) was found between mixing temperature and firmness.

Second, the adhesiveness also significantly increased (*p* < 0.05) with the increase in mixing temperature. Like hardness, the adhesiveness increased significantly from 40 to 50 °C, with its value being four times higher. No significant differences were found in samples at mixing temperatures between 30 and 40 °C and between 50 and 60 °C. A significant positive correlation (r = 0.9562) was found between mixing temperature and firmness.

Third, the spreadability of bigels increased with the increase in mixing temperature. Similar to the firmness and adhesiveness, from 40 to 50 °C its value increased significantly (*p* < 0.05); at 50 °C, the spreadability value was three times the value obtained at 40 °C. A significant negative correlation (r = 0.9619) was found between mixing temperature and firmness.

For food matrices formed by two phases, such as margarine and low-fat spreads, one of the main characteristics analyzed by consumers is their texture, i.e., their hardness and spreadability. These characteristics are determined by the composition and microstructure of the product, i.e., the shape, size, and number of crystals, and the strength of the crystalline network formed [19]. In our case, instead of fat crystals, the network’s strength is mainly due to the wax. When the bigels are mixed at a temperature near the melting point, smaller droplets are formed, as shown by microscopy, but the crystallization does not change, as shown in the XRD analyses. These findings confirm that smaller hydrogel droplets serve as fillers inside the matrix, promoting the formation of a robust and adhesive bigel that requires increased force to spread.

### 2.7. Rheology

**Figure 7:**
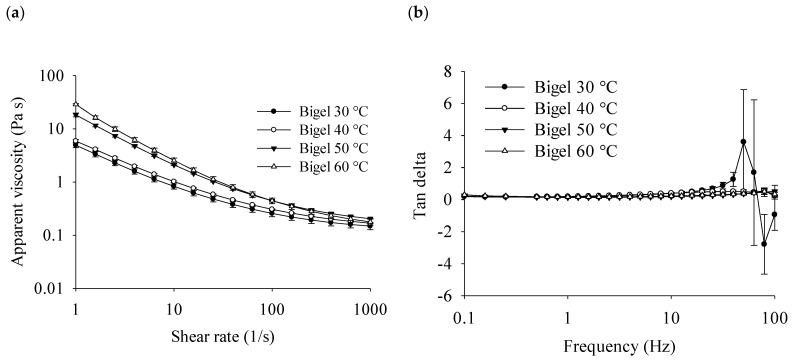

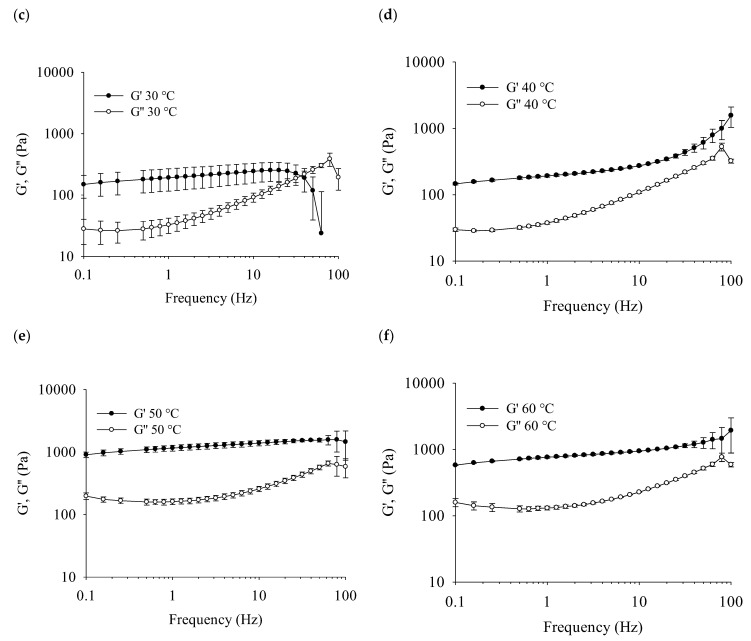
Rheological properties of bigels prepared with carnauba wax/canola oil oleogel and Arabic gum hydrogel at different mixing temperatures. Apparent viscosity (Pa⸱s) over a shear rate range from 1 to 1000 (1/s) (**a**). Changes in the viscoelastic properties tanδ (**b**), the storage modulus (G′), and loss modulus (G″) of bigels prepared at 30 °C (**c**), 40 °C (**d**), 50 °C (**e**), and 60 °C (**f**) over a frequency range from 1 to 100 Hz.

The apparent viscosity of bigels decreased when the shear rate increased (Figure 7), indicating a shear-thinning behavior similar to the carnauba wax oleogel. The shear-thinning behavior indicates a contribution to the spreadability when applied on a surface, meaning that the bigels can flow easily at higher shear rates. In contrast, at low shear rate values, the samples adopt a higher consistency. Bigels prepared at lower temperatures of 30 and 40 °C showed substantially lower viscosity than bigels prepared at 50 and 60 °C in the entire range of shear rate, suggesting that mixing temperatures close to or greater than the melting temperatures of the oleogel (Table 1) increase the mobility of the molecules constituting a greater self-assembled structure in the bigels. At shear rates under 150 (1/s), bigels prepared at 50 °C showed lower viscosity than those prepared at 60 °C; however, at higher shear rates, the viscosity of bigels prepared at 50 °C was higher. A strong positive correlation was found between apparent viscosity and spreadability, with correlation coefficients ranging from 0.863 to 0.993. The strongest correlations (>0.99) occured at shear rates between 39.8 and 99.1 (1/s), decreasing at shear rates over 630 (1/s).

The viscoelastic behavior of samples in the linear viscoelastic region showed noticeable changes when modifying the mixture temperature (Figure 7). Although at frequencies lower than 10 Hz, all bigels presented solid-like behavior with storage modules (G′) values higher than the loss modulus (G″), with slightly positive frequency dependence behavior (positive slope of moduli). At values over 10 Hz, the behavior showed differences. Bigels prepared at 30 °C exhibited a cross-over after 36 Hz, indicating that they were frequency dependent. Bigels prepared at 40 °C exhibited an increase in the slope of storage modulus after 30 Hz, which is a typical behavior for elastic networks, indicating that this mixing temperature produced bigels based on a combination of CO, CW, and AG with solid-like behavior. No crossover points were observed for the samples prepared at 50 and 60 °C, suggesting solid-like behavior with structural cohesiveness of the bigel in the entire frequency range of the linear viscoelastic region.

At 30 and 40 °C, the matrix exists in a solid-like state (below crystallization temperatures), wherein wax crystals form structures within the matrix during the preparation of bigels, which facilitates the formation of larger droplets in the bigels. The larger droplets of hydrogel cannot act as effective fillers to reinforce the matrix, so the apparent viscosities of the resulting bigels are lower and the system cannot maintain solid-like behavior at higher frequencies. In contrast, the samples prepared at 50 and 60 °C were around the crystallization point; therefore, few, very small, or no crystals were in the matrix, allowing the systems to promote the formation of small droplets. The resulting bigels can maintain a solid-like structure at high frequencies. Similarly to the findings, it has been reported that structural characteristics, together with mechanical and rheological properties, are impacted by mixing temperature; more homogeneous phase distribution has been obtained while the gels are still molten (>70 °C), while less homogeneous and stable bigels have been obtained at low temperatures (25 °C) [8].

### 2.8. Oxidative Stability

The oxidative stability of the bigels was evaluated through the induction period (IP), which measured the total time required for the oil under accelerated conditions until oxidation begins (Figure 4). In this context, the bigels showed no significant differences (*p >* 0.05) in the IP (about 8 h) when the homogenization temperature of the bigels increased from 30 to 60 °C. Consequently, while the homogenization temperature significantly influences the textural and SBC properties of bigels, it does not inherently lead to a notable improvement in oxidative stability. This suggests that once some structural integrity has been achieved, subsequent microstructure changes may not influence how quickly the bigel can initiate an oxidation reaction.

## 3. Conclusions

It was demonstrated that by modifying process parameters such as mixing temperature, the textural and SBC properties of bigels can be modulated according to the desired application. Bigels prepared at lower temperatures result in a solid-like state upon crystallization, resulting in matrices with larger hydrogel droplets, a softer texture, and lower adhesiveness, spreadability, and SBC. In contrast, bigels prepared at higher temperatures, around crystallization temperature, had no solid state and resulted in matrices with smaller hydrogel droplets with higher firmness, adhesiveness, and spreadability. Moreover, these bigels had higher apparent viscosity, particularly at lower shear rates, with solid-like behavior in the linear viscosity range. Varying the mixture temperature in the preparation of bigels did not change the thermal properties, FTIR, or oxidative stability of the samples. The results highlight the importance of hydrogel droplet size on the microstructure of formed bigels, as smaller droplets can act as effective fillers to reinforce the matrix without making chemical changes.

## 4. Materials and Methods

### 4.1. Materials

Cold-pressed canola oil was purchased from Canola de Vida (Osorno, Chile). Carnauba wax (from Copernicia prunifera) was acquired from Herbolario (Santiago, Chile). Arabic gum (from acacia tree) was acquired from Sigma Aldrich (St. Louis, MO, USA).

### 4.2. Bigel Preparation

Initially, the oleogel and hydrogel were prepared separately. After that, the two gels were mixed at different temperatures (30, 40, 50, or 60 °C) to obtain a homogenized bigel.

The oleogels were prepared by mixing the 8% (*w*/*w*) CW with CO. The mixture was heated to 90 °C and stirred (300 rpm) for 30 min; after that, the mixture was left to cool down to the required temperature. The hydrogel was prepared by mixing the AG with distilled water (4% *w*/*w*) and stirring at room temperature until complete dissolution.

Bigels were prepared by mixing the oleogel with the hydrogel in proportions of 90/10 (*w*/*w*), in batches of 100 g. The oleogel and hydrogel were mixed using a homogenizer (Benchtop, ProScientific, Oxford, CT, USA) at mixing rates of 2500 rpm for 7 min. Bigels were stored in glass containers in a refrigerator for 24 h before being analyzed.

### 4.3. X-Ray Diffraction Analysis

To examine the crystallinity of the bigels, X-ray diffraction analysis was carried out using a D8 Advance (Bruker, Karlsruhe, Germany) diffractometer equipped with a CuK_α1,2_ and K_β_ X-ray tube, set to 40 kV and 40 mA. The bigels were placed on a Lucita sample plate and gently compressed by hand. Scans were collected from 2θ = 2°–50° with step sizes of 0.02° at 1 s per step. The data were processed and analyzed using EVA software (Bruker Corporation, Billerica, MA, USA).

### 4.4. Fourier Transform Infrared (FTIR) Spectroscopy

FTIR analyses of the samples were carried out using an ATR-FTIR Spectrum One spectrometer (PerkinElmer, Waltham, MA, USA). The samples were analyzed at room temperature in a spectral range from 4000 to 400 cm^−1^ at a resolution of 4 cm^−1^, with 32 scans per sample. The spectra obtained were processed using OMNIC 9 software, including baseline correction and normalization.

### 4.5. Optical and Confocal Laser Scanning Microscopy Analyses

Optical microscopy was performed to analyze the microstructure of the samples. A Euromex optical microscope (Euromex Microscope B.V., Diuve, The Netherlands) was used, which captures images with a 100× lens.

The phase distribution of bigels was observed using an Olympus FV1000 spectral confocal microscope (Olympus Corporation, Tokyo, Japan). Nile red (30 μM in acetone) was added to stain the oil phase. The extinction/emission wavelength was set at 488/590 nm.

### 4.6. Differential Scanning Calorimetry Analysis

The thermal stability of the bigels was determined using a DSC 1 STAR (Mettler-Toledo, Greifensee, Switzerland). A mass of 20–25 mg per sample was placed in the pan, while an empty pan was used as a reference. Nitrogen gas was used as a purge gas. The samples were heated from 25 to 150 °C, then cooled from 150 to 25 °C at a scan rate of 5 °C/min. The maximum melting and crystallization temperatures were calculated using STARe software (DB V12.10, Mettler-Toledo, Greifensee, Switzerland). This analysis was performed in triplicate.

### 4.7. Solvent Binding Capacity

The solvent binding capacity (SBC) was determined using the centrifugation method to calculate the oil and/or water loss of the bigels. One gram per sample was deposited into a Falcon tube and subjected to centrifugal forces at 7000 rpm for 40 min. After that, the excess solvent was decanted onto a paper cloth, and the mass of the Falcon tube containing the residual bigel was weighed. The binding capacity (SBC) was calculated with the following equation [20,21]:

(1) $$SBC=\frac{\left[\left(m_{1}-m\right)-\left(m_{2}-m\right)\right]}{\left(m_{1}-m\right)}\times 100\%,$$

where *m* is the mass of the empty Falcon tube, *m*_1_ is the mass of the Falcon tube with the initial bigel, and *m*_2_ is the mass of the Falcon tube with the residual bigel. This analysis was conducted in triplicate.

### 4.8. Texture Analysis

To evaluate the effect of mixing temperature on the texture of bigels, a spreadability test was conducted with the TTS spreadability fixture on the texture analyzer TA.XT PlusC (Stable Micro System, Surrey, UK) and Exponent Connect software v. 7.0.6.0 (Stable Micro System, Surrey, UK). The bigels were located in a conical female probe and stored in a fridge at 6 °C for at least 6 h. To conduct the analysis, the male cone penetrated the sample in the female cone at 3 mm/s until a distance of 23 mm and a post-test speed of 10 mm/s. The hardness (maximum force), spreadability (positive area), and adhesiveness (negative area) were determined [22]. For this analysis, at least four replicates were analyzed.

### 4.9. Rheology Analysis

The viscoelastic properties of the bigels were determined using a rheometer (Discovery DHR2, TA Instruments, New Castle, DE, USA) equipped with a parallel rough plate geometry system (Plate SST ST XHatch 40 mm Smart-SW) and Peltier plate steel with an axial gap of 1000 μm, following the method described by Quilaqueo et al. [22] with some modifications. The samples were carefully covered with a solvent trap to maintain the temperature. The TRIOS software package (version 5.1.1., TA Instruments, New Castle, DE, USA) was used to control the equipment and acquire the rheological parameters. The steady shear flow measurements were taken at 25 °C in a 1–1000 s^−1^ shear rate range. The range of linear viscosity values for the samples was obtained from the plot of the elastic modulus (G′) vs. the oscillatory strain (%) under oscillatory conditions at 1 Hz, from 0.01 to 50%. In the frequency sweep test, the temperature was maintained at 25 °C, and the response of the moduli (G′, G″) to the increasing frequency (0.1 to 100 Hz) at a strain of 0.1% within the linear viscosity was measured. Three replicates of each sample were recorded for each test.

### 4.10. Oxidative Stability Analysis

The Rancimat test was used to determine the oxidative stability of the bigels. For this, 5 g of each sample was placed in the Rancimat equipment (743, Metrohm AG, Herisau, Switzerland) at 110 °C with 10 L/h of airflow (accelerated oxidation condition). The induction period (IP) was calculated from the inflection point of the conductivity curve [22]. This analysis was conducted in triplicate.

### 4.11. Statistical Analysis

A one-way analysis of variance (ANOVA) was used to determine the significant differences among the samples at a significance level of 0.05. Tukey’s test was used in cases where significant differences among the samples were found with ANOVA, using Statgraphics Centurion XV software (version 15.1.02).

## Figures and Tables

**Table 1:** Thermal properties of oleogel and bigels prepared at different mixing temperatures.

Samples	Heating		Cooling		
	T_pm 1_	T_pm 2_	T_pc 1_	T_pc 2_	T_pc 3_
Oleogel	49.8 ± 1.1 ^ab^	77.4 ± 0.1 ^ab^	54.0 ± 0.5 ^a^	53.2 ± 0.8 ^b^	38.3 ± 0.6 ^a^
Bigel 30 °C	49.1 ± 0.2 ^a^	76.7 ± 0.3 ^a^	54.6 ± 0.7 ^a^	50.9 ± 0.0 ^a^	39.8 ± 1.7 ^a^
Bigel 40 °C	49.5 ± 0.5 ^ab^	78.4 ± 2.0 ^ab^	54.7 ± 0.3 ^a^	51.6 + 0.2 ^a^	39.1 ± 0.3 ^a^
Bigel 50 °C	50.7 ± 0.5 ^b^	78.8 ± 1.3 ^ab^	54.4 ± 0.1 ^a^	51.1 + 0.3 ^a^	38.7 ± 0.2 ^a^
Bigel 60 °C	50.5 ± 0.2 ^ab^	80.0 ± 0.1 ^b^	54.3 ± 0.1 ^a^	51.1 + 0.1 ^a^	38.7 ± 0.1 ^a^

Different letters on the same column indicate statistically significant differences (*p* < 0.05); T_pm (1–2)_, peak melting; T_pc (1,2,3)_, peak crystallization.

## Data Availability

The raw data supporting the conclusions of this article will be made available by the authors on request.
